# Basic life support skills of high school students before and after cardiopulmonary resuscitation training: a longitudinal investigation

**DOI:** 10.1186/1757-7241-20-31

**Published:** 2012-04-14

**Authors:** Theresa M Meissner, Cordula Kloppe, Christoph Hanefeld

**Affiliations:** 1 Medizinische Klinik III, St. Elisabeth-Hospital, Bleichstr. 15, 44787 Bochum, Germany

**Keywords:** Basic life support, High school students, Teenagers, Cardiopulmonary resuscitation, Assessment, Retention, Sudden cardiac arrest, Automated external defibrillation

## Abstract

**Background:**

Immediate bystander cardiopulmonary resuscitation (CPR) significantly improves survival after a sudden cardiopulmonary collapse. This study assessed the basic life support (BLS) knowledge and performance of high school students before and after CPR training.

**Methods:**

This study included 132 teenagers (mean age 14.6 ± 1.4 years). Students completed a two-hour training course that provided theoretical background on sudden cardiac death (SCD) and a hands-on CPR tutorial. They were asked to perform BLS on a manikin to simulate an SCD scenario before the training. Afterwards, participants encountered the same scenario and completed a questionnaire for self-assessment of their pre- and post-training confidence. Four months later, we assessed the knowledge retention rate of the participants with a BLS performance score.

**Results:**

Before the training, 29.5% of students performed chest compressions as compared to 99.2% post-training (*P *< 0.05). At the four-month follow-up, 99% of students still performed correct chest compressions. The overall improvement, assessed by the BLS performance score, was also statistically significant (median of 4 and 10 pre- and post-training, respectively, P < 0.05). After the training, 99.2% stated that they felt confident about performing CPR, as compared to 26.9% (*P *< 0.05) before the training.

**Conclusions:**

BLS training in high school seems highly effective considering the minimal amount of previous knowledge the students possess. We observed significant improvement and a good retention rate four months after training. Increasing the number of trained students may minimize the reluctance to conduct bystander CPR and increase the number of positive outcomes after sudden cardiopulmonary collapse.

## Background

Sudden cardiac death (SCD) accounts for approximately 11% of all deaths per year in Germany and is considered a serious risk for the population [1,2]. People who suffer from sudden cardiac arrest (SCA) depend on prompt basic life support (BLS). Patients who receive bystander cardiopulmonary resuscitation (CPR) have a two to three times higher survival rate (8.2% vs. 2.5% for patients who did not receive CPR) [3]. Extensive education of the population in particular countries and regions led to high numbers of bystander CPR in cases of out-of-hospital cardiac arrests (OHCA) [3-5]. However, studies show that often less than one-third of out-of-hospital witnessed cardiac arrest victims receive bystander CPR [6,7]. Furthermore, 50-65% of cardiac arrests happen at home [8]. Because these victims are less likely to receive bystander CPR, they have poorer outcomes than those who are subject to OHCA in other non-hospital locations [9]. In these cases, bystanders are usually family members and can include high school-aged students.

Ventricular fibrillation accounts for 59-65% of analyzed rhythms that lead to SCD [10,11]. In cases of ventricular arrhythmias, defibrillation within 3-5 minutes of collapse raises the survival rate to 49-75% [12]. Defibrillation with an automated external defibrillator (AED) can be provided by a layperson before emergency services arrive and is not exceptionally difficult; it requires mostly cognitive skills and has been accomplished by school children [13].

Compared to an untrained group, bystanders with previous CPR training are more likely to perform CPR [14]. Jones et al. found that 13- to 14-year-old adolescents can perform chest compression as well as adults [15]. German teenagers, however, are already at least 17 years old when they have to undertake a compulsory first aid and BLS class in the context of acquiring their driver's license.

In Germany, there is neither legislation that assures AED installation in public places nor compulsory CPR training in high school. Nevertheless, a number of associations and independent programs advocate AED placement and are dedicated to the propagation of BLS training. One of these programs is "Bochum against sudden cardiac arrest" [16]. From this project, an emergency training and healthcare education program for young high school students (German acronym "GENOBO") was formed.

As part of the project, this study assessed the baseline knowledge of teenagers regarding BLS. Our second outcome point was to evaluate the feasibility and long-term results of the BLS training course, based on the European Resuscitation Council (ERC) guidelines [17]. For this purpose, we recorded students' improvement after the tutorial, as well as their skills retention rate four months later.

## Methods

### Study design, population and setting

This study was designed as a prospective investigation. The population included 9^th^-graders from two metropolitan high schools in the Ruhr-Area, Germany (Table 1). Parents were informed about the project by a letter and provided written consent to include their children's data in this study. The teenagers were also informed that they could refuse participation at any time.

**Table 1:** Demographic data of the participating students

Class	Number of students at the training	Boys	Girls	Mean Age	Body weight in kg	Body height in cm	BMI	First aid course	BOscore PRE-training	BOscore POST-training	BOscore Evaluation	Number of students at the Evaluation
**Total**	132	57	72	14.6 ± 1.4	58.1 ± 0.1	170.8 ± 7	19.8 ± 2.8	33.3%	3.9 ± 2.5	9.4 ± 1.1	9.4 ± 1.2	98

**1**	28	9	18	14.7 ± 2.9	59 ± 12.2	169.4 ± 6.4	20.4 ± 3.4	33.3%	3.4 ± 2.8	9.0 ± 1.1	9.1 ± 1.0	26

**2**	26	10	15	14.3 ± 0.5	55 ± 7.9	168.5 ± 7.9	19.3 ± 1.8	32%	3.6 ± 2.5	9.5 ± 0.9	9.4 ± 1.4	22

**3**	29	14	14	14.8 ± 0.5	57.7 ± 9.4	172.1 ± 6.5	19.4 ± 2.6	35.7%	4.1 ± 2.1	9.6 ± 1.0	9.5 ± 1.2	28

**4**	25	13	12	14.6 ± 0.5	61.9 ± 12.6	173.9 ± 7.2	20.5 ± 3.9	24%	4.5 ± 2.8	9.4 ± 1.2	10.0 ± 0.8	22

**5**	24	11	13	14.8 ± 0.3	56.7 ± 5.7	170.2 ± 6.1	19.6 ± 1.6	41.7%	4.3 ± 2.5	9.3 ± 1.4	-	0

This table shows the distribution of students with respect to gender, age, body weight, body height, Body-mass-index (BMI) and attendance of a first aid course prior to the training as well as with respect to attained BOscore PRE-training, BOscore POST-training and BOscore at the 4-month evaluation. The data show the mean values for each participating group and for each class separately

Teachers volunteered to take part in the project with their entire class. Each class assembled between 24 and 29 students at the initial four-hour project day, which took place at the city's emergency department. Every student was assigned an identification number. Students were individually confronted with a fake SCD scenario. The teachers guarded the not yet tested students in the first room. A Little Anne CPR Training Manikin (Laerdal Medical, Stavanger, Norway) was placed on the floor of the second room and the students were called in this testing room one after the other. There were two observers present but no other students. The observers told the student that he/she should imagine passing through the nearby park after school, when suddenly, a middle-aged runner collapsed a few meters in front of them. The teenagers were asked to act as if they were in an actual emergency and the manikin was the runner. After demonstrating all they knew to do in the given situation the students were sent into a third room, where they had the chance to talk about the scenario, first aid and health issues to paramedics, dietary experts and medical students.

Following the testing session, we explained the ideal performance of the scenario, pointing out the observed strengths and weaknesses. Then we gave age-appropriate theoretical background of SCA and BLS in about forty-five minutes. Afterwards, paramedics demonstrated the chain of survival, correct CPR performance, stable recovery position and AED application in accordance with the ERC Guidelines for Resuscitation 2010.

The students were then divided into groups of about ten people to practice CPR on a Little Anne manikin. Either a trained medical student or a paramedic supervised each group. The teenagers also practiced the correct recovery position on each other and simulated AED usage with the Lifepak CR-T AED Trainer (Medtronic) and the Laerdal HeartStart HS1 Trainer (Phillips). The effective BLS training time amounted to thirty minutes for each group of ten.

Following the practical session, the students participated in workshops on a healthy lifestyle and physical activity, which aimed at educating the teenagers on the prevention of cardiovascular diseases.

The day concluded with a re-evaluation of the students' BLS skills. They encountered the same simulated scenario and were asked to show their competencies again. We substituted the Little Anne with an ALS Skillmaster Manikin (Laerdal Medical, Stavanger, Norway).

After a four-month period, we returned to the school to evaluate the BLS performance of the students once more. The students were not aware of the scheduled assessment beforehand. The testing was conducted in the same way as immediately after the training session. This time the observers told the student to imagine being in a physical education class when a friend collapsed at the far side of the gymnasium.

### Data Collection

We documented the height, weight, age and gender of all participants. We calculated the body mass index (BMI) of the participants using height and weight (BMI = weight (kg)/height (cm)^2^).

The two observers had to agree on the student's level of BLS performance with a yes/no checklist (Table 2) in each practical assessment. The checklist we designed for this study is based on the teaching points of the CPR class.

**Table 2:** Checklist Results

Checklist item	Performed PRE-training	Performed POST-training	P-value PRE/POST	Performed at 4 months Evaluation	P-value POST/EVA
**Adress patient**	24%	92%	< 0.001	81.6%	0.06

**Shake patient**	15%	85%	< 0.001	85.7%	0.57

**Call for help**	17%	2%	< 0.001	8.2%	0.016

**Check breathing**	27%	92%	< 0.001	92.9%	1.0

**Call correct EMS number**	67%	92%	< 0.001	95.9%	0.75

**Chest compression**	29%	99%	< 0.001	99%	1.0

**Rescue breaths**	23%	92%	< 0.001	91.8%	0.39

**Median BOscore**	4 (IQR 3-6)	10 (IQR 9-10)	< 0.001	10 (IQR 9-10)	0.89

This table shows the checklist items used to assess students' performance of basic life support skills. It shows the percentage of students who performed each item at the pre-training, post-training and 4-months control. McNemar's test was used to calculate *P *values. The median BOscore resulting from this data is shown. The *P *value of the BOscore was analyzed with a paired t-test

From the items of this performance checklist, we calculated a BLS score, for an easier comparison of students' overall BLS performance. This so called BOscore (BO indicating the initials of the city of Bochum) assigned one point for each of the following items: addressing the patient, shaking the patient, calling for help, checking for breathing and mouth-to-mouth resuscitation. Calling the emergency medical services (EMS) and performing chest compressions were each awarded three points to stress the importance of these actions for survival. A maximum of eleven points was achievable.

In every session other than the pre-training hands-on session, the ALS Skillmaster software Haertsim 4000 (Laerdal Medical, Stavanger, Norway) recorded the quality, depth and frequency of compressions and the time until compressions were started. Because the 2010 guidelines were released after the project had started, the manikin first required a compression depth minimum of 4 cm. Adhering to the guidelines, it was then switched to at least 5 cm.

Students judged their BLS knowledge and confidence in their abilities before and after the training with the help of a questionnaire (Additional file 1).

### Data Analysis

Data was analyzed with IBM SPSS version 19.0 (IBM Corporation, New York, United States of America). For continuous data, we reported mean values with standard deviations and medians with inter quartile ranges (25-75% IQR). We used a paired t-test for comparison and the Mann-Whitney-U rank sum test for a comparison of groups. Absolute numbers and percentages described categorical data. The McNemar and X^2^-test were used for comparison. An independent sample t-test analyzed the differences between groups. Regression models assessed the effect of age, height, weight, BMI and gender on outcomes. All checklist data were dichotomized. The chest compression quality reported by the manikin included compression depth, frequency of compression and time until the compressions were started. *P *values < 0.05 were considered statistically significant and were all two-sided.

## Results

We trained 132 high school students. The questionnaire was completed by 129 students. Among those, 57 were boys and 72 girls. The mean age was 14.6 ± 1.4 years, with no significant difference between the mean age of the boys and girls (14.8 vs. 14.5 years, *P *= 0.21). One-third of students had attended a first aid course before. The difference in performance of those who had versus those who had not had former first aid training was only slightly significant (*P *= 0.049).

At the pre-training assessment, the median BOscore was 4 and ranged from 0-10 points (IQR 3-6). The post-training median score was 10, ranging from 5 to 11 (IQR 9-10). For each BLS item, *P *values of the pre-/post-training comparison were inferior to 0.05 and therefore considered statistically significant (Table 2).

The Skillmaster Manikin in the post-training assessment recorded data on chest compression quality for 103 participants (Table 3). The mean frequency of chest compressions was 99.3 ± 23 per minute (median = 97, min = 11, max = 164, IQR = 85-115). The required compression depth was not attained by 18%, and only 10% achieved it 100% of the time. Students achieved a median compression depth of 4-5 cm with a range of 3-6 cm (IQR 3-5 cm).

**Table 3:** Chest compression data

Item	Mean Frequency of chest compressions per minute		Mean compression depth in cm		% of students attaining a depth of 5 cm at ≥ 50% of compressions		Mean time until the start of chest compression in seconds	
	***POST-Training***	***at 4 months***	***POST-training***	***at 4 months***	***POST-training***	***at 4 months***	***POST-training***	***at 4 months***

**Total**	99.3 ± 23	102.4 ± 21.8	4.8 ± 0.5	4.9 ± 0.6	54,3	47,3	34.5 ± 16.8	41.8 ± 23.1

**Boys**	100.1 ± 23.5	105.3 ± 19.7	5 ± 0.6	5.2 ± 0.6	57,4	50,9	35.4 ± 17.9	42.2 ± 21.1

**Girls**	97.9 ± 21.4	100.5 ± 23.3	4.6 ± 0.5	4.7 ± 0.6	50,3	41,8	33.1 ± 15.8	42.0 ± 25.2

Data was calculated from the recordings of the Skillmaster Manikin

The mean time until the start of chest compressions was 35 ± 17 seconds (median = 33, min = 7, max = 117, IQR = 24-41).

Table 4 shows the results from the questionnaire. Students who had considered themselves apt to apply CPR before the training did not achieve a significantly higher pre-training BOscore (4.3 vs. 3.9, *P *= 0.47).

**Table 4:** Self-confidence assessment

Item	Percentage of students that checked "yes"		
	***Stable recovery position***	***CPR***	***AED***

**Knew about**	93.8%	90.8%	90.8%

**Would have dared to apply** **the following measure before the** **training**	95%	26.9%	15%

**Would dare to apply the following** **measure on a person in need the day** **after the training**	46%	99.2%	87%

**Attended first-aid training before**	33.3%		

This data on the students' self-confidence was taken from the questionnaire [see Additional file 1] filled out privately by each participant at the end of the training day

The evaluation after four months included 98 students (Table 1). The median BOscore was equal to the post-training score (Table 2). There was no statistically significant difference between the BLS checklist items other than the call for help. Interestingly, in the four-month assessment, students called for help more frequently than directly after the training (1.5% vs. 8.2%, *P *= 0.01).

We observed no differences between the overall BLS performance of boys and girls. The *P *values comparing BOscores were 0.90 for the pre-training, 0.56 for the post-training and 0.24 for the evaluation after four months. Boys had a wider interquartile range in the pre-training score (IQR: pre-training BOscore for boys 1-6 points, 3-5 points for girls). The mean learning effect was also not related to gender; boys and girls improved with 5.3 ± 2.8 and 5.5 ± 2.7 points, respectively.

However, there was a significant difference in the depth of chest compressions depending on gender. Although the median compression depth for both groups was 4-5 cm, more boys than girls achieved compressions between 5 and 6 cm (18.2% vs. 1.8%, *P *= 0.001). This might partially be because the boys were significantly taller (175.2 ± 6.7 cm vs. 167.5 ± 5.1 cm, *P *< 0.001) and heavier (62.8 ± 11.7 kg vs. 54.4 ± 6.7 kg, *P *< 0.001) than their female classmates. Surprisingly a closer analysis showed no correlation between body weight, height, BMI or age and the depth of chest compressions. We reasoned that there must be some other variable involved. Deducing from our observations during the training as well as from the fact that significantly more boys (28.1%) than girls (5.6%) had judged themselves confident with AED use before the training (P < 0.001), we can state that the majority of the girls might be more cautious than their male peers - not only in their self-evaluation, but also in their performance.

## Discussion

SCD is the most common cause of death worldwide. It affects about 350,000 to 700,000 people in Europe annually and its incidence is expected to rise in the coming years [18].

Although the SCA incidence at schools accounts for only 2.6% of all public location SCAs [19], a trained student could witness a medical emergency that requires CPR in any location. According to the Teenmark Survey 2003 [20], American teenagers (12-17 years of age) spend a good amount of their free time in shopping malls. Becker et al. reported that large shopping malls are public locations with a high incidence of cardiac arrests (ten in five years) [21]. 35 and 11 cardiac arrests per site were registered in international airports and public sports venues, respectively, over five years [21].

Earlier studies have shown that even nine-year-old school children have the cognitive skills to perform CPR after specific training [22,23]. The limiting factor for these children to perform adult CPR correctly is their body mass. Students who can attain a compression depth of at least 4 cm can resuscitate other children [24]. In accordance with the science advisory of the American Heart Association [25], we chose giving courses to 9th graders. Younger students might have been discouraged or disinterested in CPR, resulting from a physical incapability to deliver high-quality CPR.

**Figure 1:**
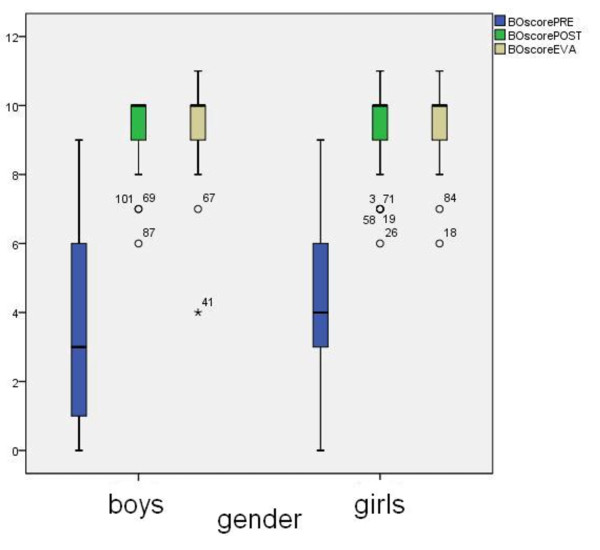
**lllustration of the overall basic life support performance of teenagers**.

We found that the teenagers' baseline BLS skills were poor when they entered the training. We saw significant improvement after our theoretical and practical training course. BLS skills after four months were at nearly the same level as directly after training (Figure 1).

The psychological phenomenon of the "bystander effect" occurs when someone witnesses an emergency but does not help the victim because other people are present. It is often observed in emergencies [26]. Students who feel competent and understand the concept of "diffusion of responsibility" will also be more likely to help [27-29]. Furthermore, Roppolo et al. stated that early CPR training contributes to better retention rates for subsequent courses [30]. We also hope to decrease the most common fears associated with CPR. Explaining the theoretical background on SCA makes students understand the necessity of providing immediate CPR and reduces the reluctance to help in a critical situation.

BLS and AED training in schools should emphasize the recognition of an emergency and the provision of high-quality chest compressions [31,32]. Our BLS training also focused on these two aspects. We stressed the concept of agonal breathing (gasping) because it is important that students do not mistake this as normal breathing, which would prevent them from starting CPR. If adolescents know how to identify a dangerous situation, they can interpret a situation as an emergency and provide at least indirect help by calling the ambulance, for example.

Concerning AED usage, our main goal was to explain its purpose to the teenagers. Ventricular arrhythmias are typically the cause of sudden cardiac arrest and are more common than severe bradycardia or pulseless electrical activity [18]. For each minute that passes before defibrillation, the chance of survival is reduced by about 10-12% [33]. We did not assess the correct deployment of an AED in the control sessions because earlier studies showed that even elementary school students already have the cognitive skills to correctly apply an AED [34].

The depth, rate and interruption duration of the compressions directly influence the outcome of cardiac arrest [35,36]. Those quality-determining factors are best learned through practice [37]. Although other studies advocate the use of video-based self-training kits to reach a larger number of people and reduce costs [38,39] we favored hands-on training within small groups led by skilled instructors, who can give individual feedback and advice.

We did not use the Skillmaster Manikin for the first assessment of compressions because we saw that the vast majority of students in pre-study testing did not perform correct CPR. Therefore, we thought that the expensive manikin would not be advantageous in the pre-training examination because it would yield limited, if any, useful data.

Putting the patient in a stable recovery position was only assessed in the pre-training session. After the training, all students correctly realized that the fake scenario required CPR; consequently, the item had become irrelevant.

There were two unexpected results in our study. First, we found that fewer students than before the training called for help in the post-training assessment. This may be due to the fact that students were focused on performing CPR. We strongly emphasized the importance of chest compressions in the training. Before the training, most students were unsure of how to act in an emergency and reacted "naturally" by calling for help. We assume that even a trained student would become insecure in a real emergency and call for help before starting CPR.

Second, fewer students reported confidence in putting the patient in a stable recovery position after the training than before, which we found to be astonishing. Despite, or perhaps because of the hands-on practice of the stable recovery position recommended by the AHA, it seems that students perceived the position as a very complex task. This needs to be considered and prevented in future training sessions.

Students that had already received first aid training in primary school did not perform better in the pre-training than their peers. Interviewing the responsible persons showed that the training had focused on the social responsibility of helping others. There was hands-on training for the stable recovery position and bandaging. CPR was only briefly demonstrated.

Educating school children about BLS is an excellent strategy to reach a broad public and increases the percentage of trained adults in a community [31]. If students share the acquired knowledge with their families and non-trained friends, we indirectly introduce a larger part of the community to the topic of BLS and AED deployment [30]. This raises the likelihood that a trained person will be present at an SCA event scene to perform immediate bystander CPR.

### Limitations

The high schools that participated offer only honors classes. Students at those schools are considered to have a readiness of mind and good comprehension. In addition, the schools are situated in a middle-class neighborhood. These two factors may lead to a selection bias for the learning effect and retention capacities.

Absence of students due to scheduling issues, as well as class turnover and organizational difficulties on the schools' side, led to a reduction of the data at the four-months' evaluation (Table 1, group 5).

Due to a technical problem, post-training chest compression data of 26 students were incomplete.

The BOscore is not yet validated, because it was specifically designed for this study and was used here for the first time.

## Conclusions

The fundamental BLS knowledge of high school students was meager. We have demonstrated that teenagers' acquisition of BLS/CPR knowledge is adequate and that skill retention over four months is stable. As shown previously, high school students have the cognitive and physical ability to act as first bystanders in an emergency by providing CPR to children and even adults. We share the opinion that schools are an ideal setting to teach BLS and CPR skills because a large part of the community is introduced to these life-supporting prospects. In accordance with previous suggestions, we highly recommend that BLS/CPR modules are implemented as a mandatory part of the physical education or science curriculum in Germany.

## Abbreviations

AED: Automated external defibrillator; AHA: American heart association; BLS: Basic life support; BMI: Body-mass index; CPR: Cardiopulmonary resuscitation; EMS: Emergency medical services; ERC: European resuscitation council; GENOBO: Gesundheitsförderung und notfalltraining an bochumer schulen; IQR: Inter quartile range; OHCA: Out-of-hospital cardiac arrest; SCA: Sudden cardiac arrest; SCD: Sudden cardiac death

## Competing interests

The GENOBO project received a one-time financial grant by the Medtronic Foundation. The local incorporated Society of Cardiology funded some of the training day materials. No authors have any financial or personal relationships with the organizations subsidizing this project.

## Authors' contributions

All authors have made substantial contributions to the conception and design of the study, analysis and interpretation of data, drafting the article and revising it critically for important intellectual content. CH initiated the project, supervised it, and gave administrative, technical and material support. TM collected the data. All authors read and approved the final manuscript.

## Supplementary Material

Additional file 1 **Questionnaire**. Click here for file
