# Supplementary material for: Basic life support skills of high school students before and after cardiopulmonary resuscitation training: a longitudinal investigation
Source: Scand J Trauma Resusc Emerg Med. 2012 Apr 14;20:31. doi: 10.1186/1757-7241-20-31 (PMC3353161; doi:10.1186/1757-7241-20-31)
Supplement: Additional file 1 — Questionnaire. [file 1757-7241-20-31-S1.DOCX]

**Additional file 1- Questionnaire**

This table shows the questions that each student answered privately to assess demographic data and self-confidence.

| **Item** | **If you can answer the question with „yes“, check the corresponding box, please.** | | | | | |
| --- | --- | --- | --- | --- | --- | --- |
|  | *Stable recovery position* | | *CPR* | | *AED* | |
| **Did you know about any of these measures before you came to this training day?** |  | |  | |  | |
| **Did you know how to carry out any of the named measures correctly?** |  | |  | |  | |
| **Would you have dared to apply any of those measures before the training?** |  | |  | |  | |
| **Would you dare to apply any of those measures if you found a person in need of it tomorrow?** |  | |  | |  | |
| **Did you attend a first-aid training before?** | *at school* |  | *in kindergarden* |  | *elsewhere (please specify)* | |
| **When did that training take place?** |  | | | | | |
| **How old are you?** | *years* | | | | | |
| **How tall are you?** | *cm* | | | | | |
| **How much do you weigh?** | *kg* | | | | | |
| **Are you a …** | *boy* |  | *or a ..* | | *girl* |  |
